# Biofilm Modifies Expression of Ribonucleotide Reductase Genes in *Escherichia coli*

**DOI:** 10.1371/journal.pone.0046350

**Published:** 2012-09-26

**Authors:** Maria del Mar Cendra, Antonio Juárez, Eduard Torrents

**Affiliations:** 1 Cellular Biotechnology, Institute for Bioengineering of Catalonia (IBEC), Barcelona, Spain; 2 Department of Microbiology, Facultat de Biologia, Universitat de Barcelona, Barcelona, Spain; University of Würzburg, Germany

## Abstract

Ribonucleotide reductase (RNR) is an essential enzyme for all living organisms since is the responsible for the last step in the synthesis of the four deoxyribonucleotides (dNTPs) necessary for DNA replication and repair. In this work, we have investigated the expression of the three-RNR classes (Ia, Ib and III) during *Escherichia coli* biofilm formation. We show the temporal and spatial importance of class Ib and III RNRs during this process in two different *E. coli* wild-type strains, the commensal MG1655 and the enteropathogenic and virulent E2348/69, the prototype for the enteropathogenic *E. coli* (EPEC). We have established that class Ib RNR, so far considered cryptic, play and important role during biofilm formation. The implication of this RNR class under the specific growth conditions of biofilm formation is discussed.

## Introduction

*Escherichia coli* biofilm development is a complex molecular process that involves a large number of genetic factors and genes. When the global gene expression profiles of biofilm and planktonic *E. coli* cells are compared, very significant differences are apparent [1]. Biofilm formation is underlying catheter-associated urinary tract infections (CAUTIs), urinary tract infections (UTIs) caused by uropathogenic *Escherichia coli* (UPEC), and the various types of diarrhea caused by enterohemorrhagic, enteroinvasive and enteroaggregative *E. coli.* The persistence of these biofilms may contribute to such infections becoming chronic conditions [2].

The complexity of biofilm formation makes it difficult to precisely identify the regulatory networks and the processes of alteration of gene expression which account for its development. In this context, it is important to understand how DNA synthesis is regulated and which factors participate in this process.

Although *E. coli* K-12 is not as proficient in making biofilms as other *E. coli* isolates, it has nonetheless been used in many studies, for instance to elucidate changes in transcriptomic profiles between planktonic cells and biofilm [1], [3], [4], [5]. Confocal analysis has evidenced a well-defined three-dimensional colonial structure with a mushroom form in K-12 strains and also the enteroaggregative E2348/69 *E. coli* strain [3], [6].

Ribonucleotide reductases (RNR) are essential enzymes in all living cells. These proteins catalyze the reduction of ribonucleotides (NTPs) to the corresponding deoxyribonucleotides (dNTPs), thus providing the buildings blocks for DNA synthesis and repair [7]. The three known RNR classes (I, II and III) use free radicals for catalysis but rely on different metallocofactors for the initiation of the radical reduction process, each exhibiting a different behavior towards oxygen [8]. Class I RNRs contain a stable tyrosyl radical and an oxygen-linked di-iron center required for the production of free radicals, and class I enzymes are only functional under aerobic conditions. This class of RNRs is further subdivided into classes Ia and Ib, because of significant differences in allosteric regulation and gene organization. Class Ia RNRs are encoded by the *nrdA-nrdB* genes, whereas class Ib RNRs are encoded by the *nrdH-nrdI-nrdE-nrdF* genes. It has been recently shown that class Ib small subunit (NrdF) stabilizes the tyrosyl radical by a dimanganese-oxo center [9], [10].Class II RNRs are coenzyme B12-dependent and can be active under both aerobic and anaerobic conditions. Lastly, class III RNRs, encoded by the *nrdD-nrdG* genes, carry a stable but oxygen-sensitive glycyl radical, and are only functional under anaerobic conditions. While almost all eukaryotic organisms encode exclusively class I RNRs, prokaryotes are known to encode more than one RNR class [8]. And additional protein, termed NrdR, was recently described as a novel transcriptional regulator capable of modulating the expression of all RNRs present in one organism [11].

*E. coli* and all enterobacteria encode three different RNR classes in their genome: Ia, Ib and III. While in *E. coli* class Ia RNR is active during aerobiosis and class III is active during anaerobiosis [12], [13], class Ib RNR has been considered a cryptic enzyme with no apparent function [7], [14]. However, a physiological role during growth of *E. coli* under iron starvation conditions has been assigned to class Ib. This enzyme requires manganese for tyrosyl radical generation and can replace the iron-dependent class Ia RNR [15]. A yet unanswered question is why *E. coli* encodes so many different RNRs which are apparently redundant. A hypothesis to be tested is that the different RNR classes present in *E. coli* are differentially expressed when cells deal with specific environmental signals, such as those found within the host or during biofilm formation. In this paper we evidence that when *E. coli* cells develop biofilms, expression of the different RNR enzymes is different to that observed in planktonic cells.

## Results and Discussion

### *E. coli* class Ib (*nrdE*) and class III (*nrdD*) RNR genes are key enzymes in biofilm formation

In this work we have used two different *E. coli* wild-type strains: the commensal MG1655 [16] and the enteropathogenic (EPEC) and virulent E2348/69 [17], this latter being the prototype for the EPEC *E. coli* strains involved in human disease and that still remains as the leading cause of infantile diarrhea in developing countries. EPEC colonize the proximal small intestine, where they adhere to epithelial cells forming microcolonies. Typically, EPEC strains form biofilms more complex in structure than MG1655 [6], [17]. Since class Ia RNR mutants are non-viable under normal laboratory growth conditions [14] we have studied only class Ib (*ΔnrdE*) and III (Δ*nrdD*) mutants. These mutants have been used to assay surface-associated biofilm formation on polyvinyl chloride (PVC) 96-well plates as described in the methods section.

**Figure 1:**
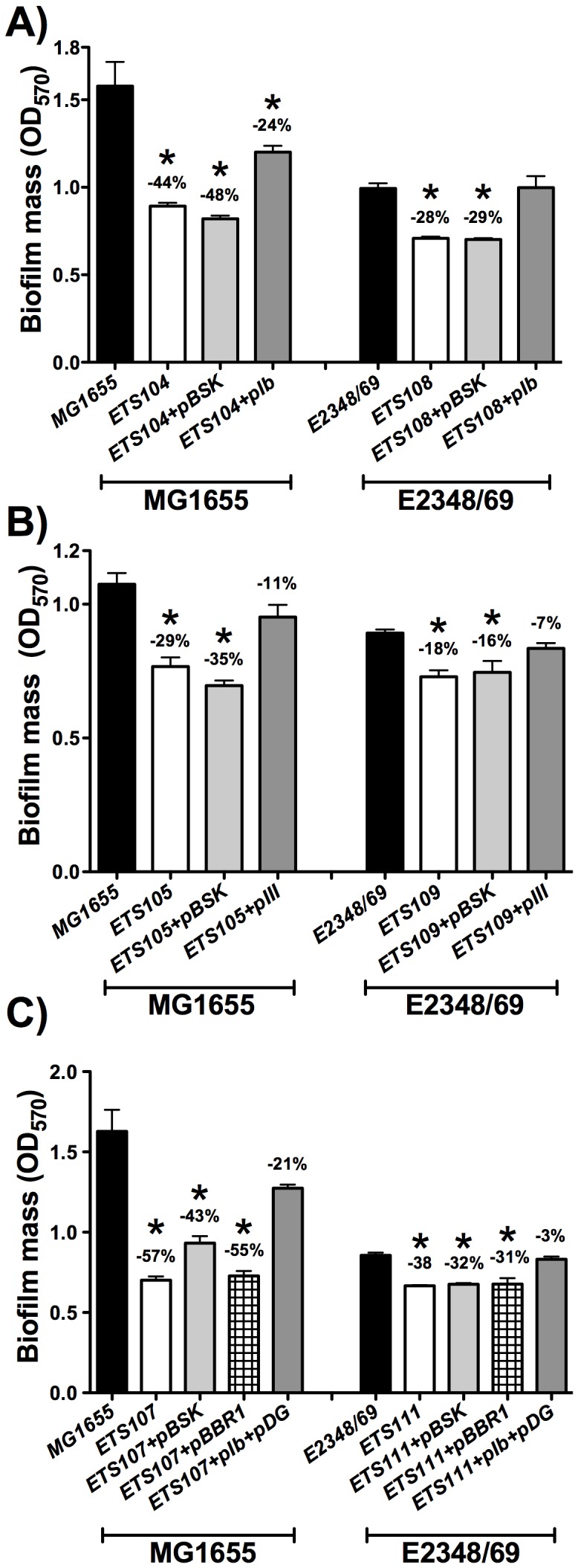
The *Escherichia coli ΔnrdE* and *ΔnrdD* mutants are defective in biofilm formation. Biofilms of the wild-type *E. coli* strains (MG1655 and E2348/69) are compared to their isogenic *ΔnrdE* mutant in A), Δ*nrdD* mutant in B) and Δ*nrdE*Δ*nrdD* double mutant in C). The values shown are the means of at least 4–5 independent experiments with six wells in each and the error bars represent standard deviations. Differences with respect to the wild-type strain were statistically significant for all the pairs of strains (*, P<0.05 by the Mann-Whitney test). Plots and statistics were generated using GraphPad Prism 5.0 software.

Deletion of the *nrdE* gene in strains MG1655 and E2348/69 (ETS104 and ETS108) reduced by about 44% and 28% biofilm formation with respect to parental wild-type strains (Fig.1A). Quantification of viable cells in the Δ*nrdE* mutant revealed a 13.6% and 20.1% increase of planktonic cells when compared to parental wild-type strains, thus corroborating the noted decrease in biofilm formation (data not shown).

With respect to the *nrdD* gene, mutants lacking this enzyme (ETS105 and ETS109) produced in both strains 29% less biofilm formation than the corresponding parental wild-type strains (Fig.1B). To correlate loss of function of *nrdD* and *nrdE* genes and alterations in biofilm formation, these mutations were complemented with the corresponding wild-type alleles and biofilm formation was tested in these constructs. When plasmids containing the *nrdHIEF* and *nrdDG* operon (pIb and pIII) were transformed in the ETS104/ETS108 (Fig.1A) and ETS105/ETS109 strains (Fig.1B), the biofilm formation level was restored to levels comparable to those observed in the corresponding wild-type strains (MG1655 and E2348/69). We decided next to combine the two mutations. A double Δ*nrdE*Δ*nrdD* mutation (ETS107 and ETS111) produced biofilm formation levels that were 57% and 38% lower than the wild-type strains (Fig.1C).

**Figure 2:**
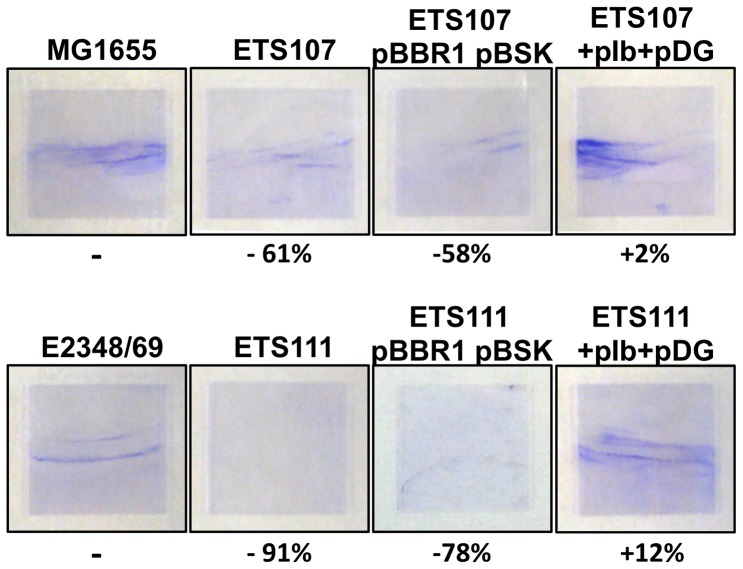
Biofilm formation in glass surface also shows that double Δ*nrdE*Δ*nrdD* mutants are defective in biofilm formation. Biofilm formation was compared in wild-type *E. coli* MG1655 and E2348/69 strains to their isogenic double Δ*nrdE* and Δ*nrdD* mutants. Complementation experiment and controls are also shown.

The absorbance levels of biofilm formation on a microtitter plate of strains MG1655 and E2348/69 are not high, but they proved to be highly reproducible. To corroborate the microtiter biofilm formation data, we performed additional assays in a glass tube, testing thus biofilm formation on a different surface. Fig.2 shows the biofilm formation of both wild-type *E. coli* strains and their corresponding double *ΔnrdE* and *ΔnrdD* mutant derivatives growing under aerobic conditions. Simultaneous deletion of the *nrdE* and *nrdD* genes in strains MG1655 or E2348/69 results either in a drastic reduction of biofilm (61% in strain ETS107) or almost completely abolishing it (91% in strain ETS111). When the double mutants ETS107 and ETS111 were complemented with plasmids pIb and pDG the biofilm formation level was restored to levels comparable to those of the corresponding parent strains (MG1655 and E2348/69). Glass tube biofilm formation was also checked by growing the different strains under microaerophlic conditions but significant biofilm growth could not be observed (data not shown).

All these results evidence that both class Ib and class III RNRs play a significant role during *E. coli* biofilm formation.

### The transcriptional factor *nrdR* induces biofilm formation through the activation of class Ib and III genes

**Figure 3:**
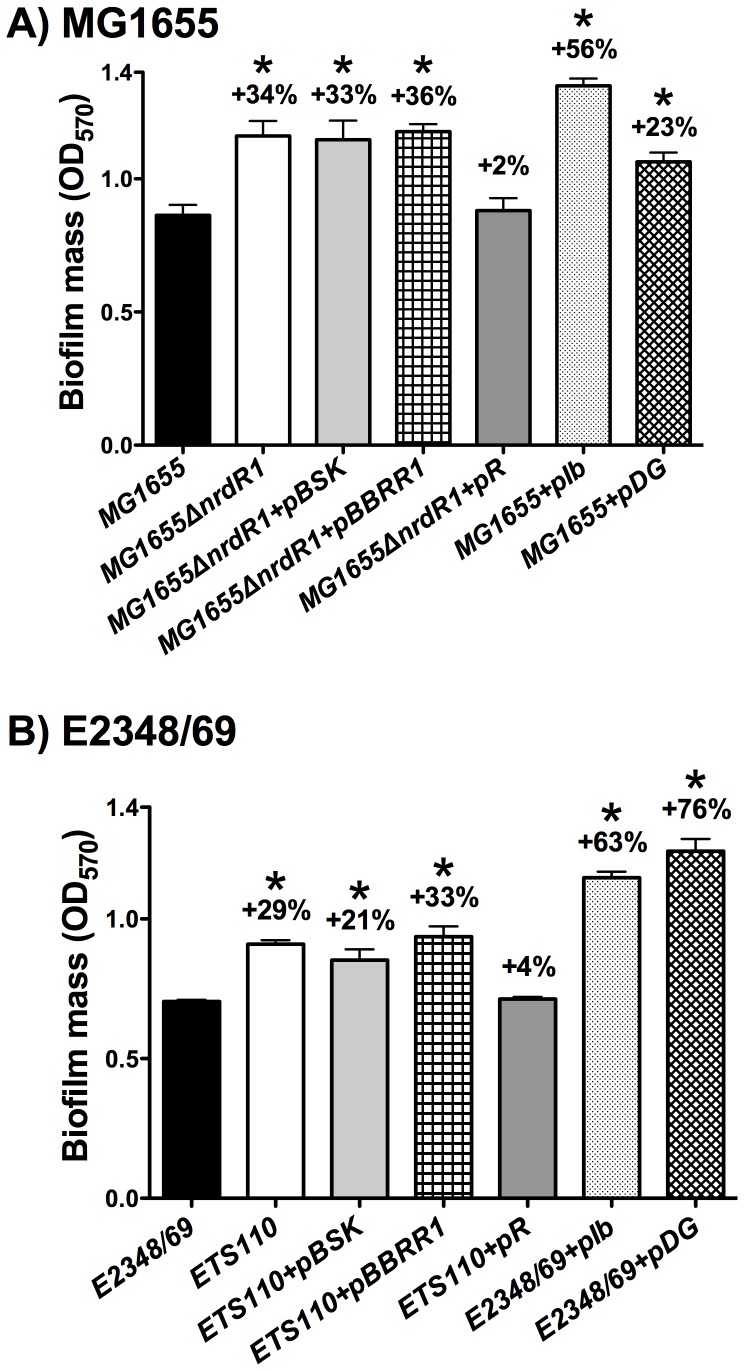
The *E. coli* Δ*nrdR* mutant enhances biofilm formation through the class Ib and III RNR classes. Biofilms of the wild-type *E. coli* strains MG1655 in panel A and E2348/69 in panel B are compared to their isogenic *ΔnrdR* mutant. The values shown are the means of at least 7–8 independent experiments with six wells in each and the error bars represent standard deviations. Differences with respect to the wild-type strain were statistically significant for all the pairs of strains (*, P<0.05 by the Mann-Whitney test). Plots and statistics were generated using GraphPad Prism 5.0 software.

The transcriptional factor NrdR acts as a repressor of the three-*nrd* genes in *E. coli* [11]. We decided to test if this protein plays a role in biofilm formation. To this purpose, *ΔnrdR* mutant derivatives were constructed in both MG1655 and E2348/69 strains. When compared to the corresponding parent strains, biofilm formation was higher (by 34% and 29%) in the Δ*nrdR* mutant derivatives (MG1655ΔnrdR1 and ETS110) (Fig3a and 3b). This suggests *nrdE* and *nrdD* up-regulation in Δ*nrdR* cells. Furthermore, a complementation test using pR plasmid (Table1) restored the biofilm levels to those of the wild-type strains. With respect to the mechanism by which NrdR enhances *E. coli* biofilm formation, it should be commented here that it had previously been shown that NrdR deficiency strongly increases class Ib RNR transcription from 25 to 50 times and class III RNR from 6 to 10 times in strain MG1655 depending on the growth phase [11]. We decided to measure transcription of *nrdE* in *E. coli* E2348/69strain and verify the results in the MG1655 strain by RT-PCR in cells grown at OD_550_ = 0.2 and 0.8. The expression level of the *nrdE* (class Ib) gene resulted to be 15.24 and 23.84 fold higher (means of three different experiments) in the E2348/69Δ*nrdR* mutant compared to the wild-type E2348/69 cells. To test whether the increased biofilm formation in an *nrdR* mutant was due either to class Ib or III RNR, we independently overexpressed the entire class Ib operon (*nrdHIEF*) and class III operon (*nrdDG*) in wild-type strains MG1655 and E2348/69 by using plasmids pIb and pIII (see Table1). We found a 56% and 63% higher level of biofilm formation compared to that produced with the Δ*nrdR* mutant when class Ib RNR was overproduced and 23% and 76% when is overproduced class III RNR. Again, these results corroborate the importance of both RNR classes (Ib and III) during biofilm formation.

**Table 1:** *Strains, plasmids and bacteriophages used in this study.*

Strain or plasmids	Description	Source
**Plasmids**		
pGEM-T easy	A/T cloning vector (Amp^R^)	Promega
pBluescriptSK	High-copy number cloning vector (Amp^R^)	Stratagene
pBBR1MCS-5	High-copy number cloning vector (Gm^R^)	[36]
pETS130-GFP	Promoterless GFP (Gm^R^)	[34]
pIb	*nrdHIEF* operon cloned into BamHI site of pBluescriptSK(+)	This work
pIII	*nrdDG* operon cloned into BamHI site of pBluescriptSK(+)	This work
pR	*nrdR* cloned into BamHI site of pBluescriptSK(+)	This work
pDG	*nrdDG* operon cloned into BamHI site pBBR1MCS-5	This work
pETS150	pETS130 derivative carrying *nrdA* promoter	This work
pETS151	pETS130 derivative carrying *nrdH* promoter	This work
pETS152	pETS130 derivative carrying *nrdD* promoter	This work
		
**Strains**		
DH5α	*recA1 endA1 hsdR17 supE44 thi-1 relA1 Δ(lacZYA-argF)U169 deoR Φ80dlacZM15*	Laboratory stock
JW2650	*E. coli* K-12 strain BW25113 Δ*nrdE::kan* (Kan^R^)	[30]
JW4197	*E. coli* K-12 strain BW25113 Δ*nrdD::kan* (Kan^R^)	[30]
JW5437	*E. coli* K-12 strain BW25113 Δ*rpoS::kan* (Kan^R^)	[30]
UA6068	*E. coli* MC1061 Δ*nrdD:: Cm* (Cm^R^)	[14]
MG1655	*E. coli* MG1655 wild type	
MG1655*ΔnrdR1*	*E. coli* MG1655 with a HpaI-Bsp119I *nrdR* deletion	[11]
ETS104	*E. coli* MG1655 Δ*nrdE::kan* (Kan^R^)	This work
ETS105	*E. coli* MG1655 Δ*nrdD::kan* (Kan^R^)	This work
ETS106	*E. coli* MG1655 Δ*nrdR::kan* (Kan^R^)	This work
ETS107	*E. coli* MG1655 Δ*nrdE::kan* (Kan^R^) Δ*nrdD::Cm* (Cm^R^)	This work
ETS113	*E. coli* MG1655 Δ*rpoS::kan* (Kan^R^)	This work
E2348/69	*E. coli* 0127:H6 E2348/69 wild type enteropathogenic	[17]
ETS108	*E. coli* E2348/69 Δ*nrdE::kan* (Kan^R^)	This work
ETS109	*E. coli* E2348/69 Δ*nrdD::kan* (Kan^R^)	This work
ETS110	*E. coli* E2348/69 Δ*nrdR::kan* (Kan^R^)	This work
ETS111	*E. coli* E2348/69 Δ*nrdE::kan* (Kan^R^) Δ*nrdD::Cm* (Cm^R^)	This work
ETS112	*E. coli* E2348/69 Δ*rpoS::kan* (Kan^R^)	This work
		
**Phagues**		
P1vir Phage		Laboratory stock

### Differential expression of *E. coli* RNR genes during biofilm formation

**Figure 4:**
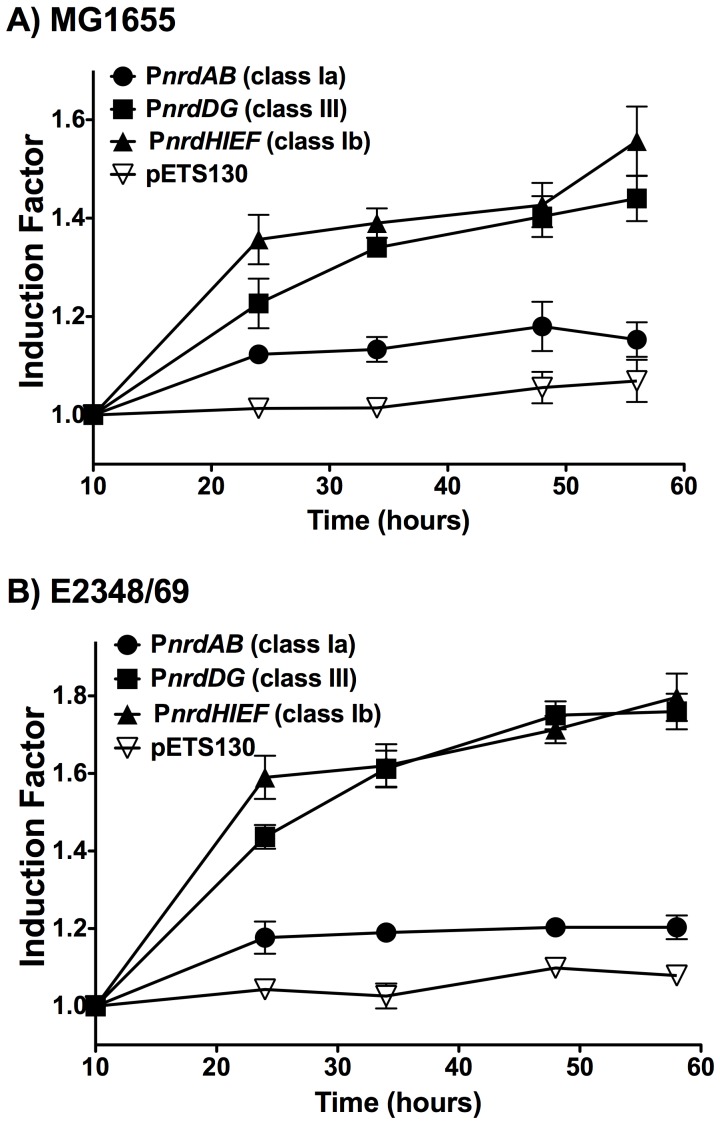
Expression of *E. coli* RNR classes during biofilm formation. Curves show fold changes expression of the different RNR promoter classes (*PnrdAB* – class Ia; *PnrdDG* – class III; *PnrdHIEF* – class Ib; pETS130 as a empty vector) from different times points during the biofilm formation of MG1655 (A) and E2348/69 (B) *E. coli* strains. All values are normalized respect the expression detected in biofilm after 10 h. The values shown are the means of three independent experiments with six wells in each and the error bars represent standard deviations.

Previously published data (NCBI GEO dataset; GDS2768, GDS2753, GSE18362, GSE13418) have rendered contradictory results with respect to the differential expression of the *nrd* genes in *E. coli* biofilms *vs.* planktonic cells: some of the *nrd* genes have been found to be up-regulated in some studies and down-regulated in others. Due to these contradictory observations we decided to carry out gene expression analysis of each *nrd* promoter during the course of the biofilm formation. We used *E. coli* MG1655 and E2348/69 cells transformed with plasmids carrying the transcriptional fusion of each *nrd* promoter to the *green fluorescent protein* (pETS150, pETS151 and pETS152) and the control plasmid pETS130 as described in the material and methods section. Fig.4 shows induction of expression of different *nrd* genes indicated as a fold-change compared to the 10 h biofilm formation. In this experiments we clearly see the *in vivo* RNR class expression shift during the course of biofilm formation. We observed that *E. coli* MG1655 (Fig.4A) and E2348/69 (Fig.4B) exhibited the lowest expression of *PnrdAB* along the biofilm formation. However, the P*nrdHIEF* operon exhibited the highest increase of expression in the first hours of the biofilm formation. In a mature biofilm (≈55 h) both the P*nrdHIEF* and P*nrdDG* construction are significantly induced (1.6 and 1.8 fold induction in the *E. coli* MG1655 and E2348/69 respectively) and (1.4 and 1.8 fold induction in the *E.* coli MG1655 and E2348/69 respectively). In contrast the expression of the P*nrdAB* promoter remained the same along the time, thus demonstrating the scarce importance of this operon during the biofilm formation. Control plasmid did not show any expression variation along the experiment.

**Figure 5:**
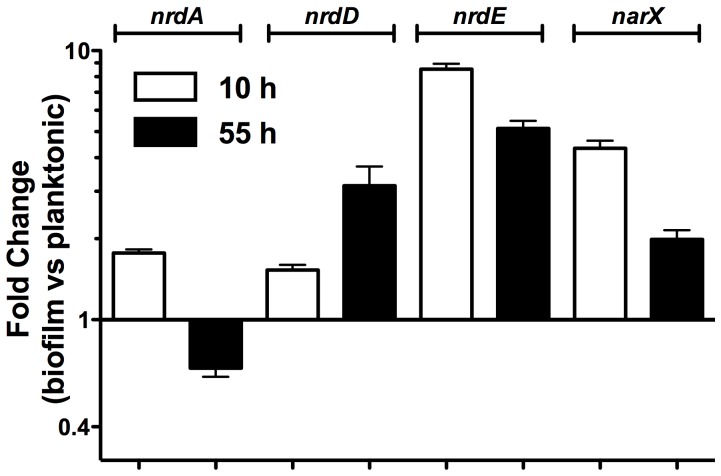
Relative expression ratio of RNR genes in biofilm *versus* planktonic cells. The expression level of the indicated genes was measured in cDNA samples derived from 10- and 55-h old *E. coli* MG1655 biofilms grown at 37°C in PVC plates. For each gene the relative expression was defined as the level in cells from biofilm compared to those from planktonic cells. The 16S rRNA sequence was used as an internal standard. Error bars represent the SD from n = 3 independent replicates.

To corroborate these data we measured expression of *nrdA, nrdD* and *nrdE* by real-time PCR with probes from *nrdA*, *nrdD* and *nrdE* [11] in RNA samples extracted from biofilms and from planktonic MG1655 *E. coli* cells grown for 10 h and 55 h. Figure5 shows the differential expression pattern for each of the *nrd* genes. Biofilm formation led to *nrdA* (class Ia) gene repression along the biofilm formation (1.55 fold repression in cells collected at 55 h). In contrast, when compared to the level in planktonic cells, expression of *nrdE* (class Ib) and *nrdD* (class III) was higher in biofilm forming cells (from 1.5- to 9-fold). The highest expression level takes place in the *nrdE* gene at any time interval. It is important to note that the expression level of the *nrdD* gene is moderate (only 1.5 fold higher) at the beginning of biofilm formation (10 h) but increases considerably (to 3.2 fold) in a mature biofilm (55 h) in which there are areas where conditions are microaerophilic. To confirm microaerophilic conditions, expression of the *narX* gene, which is controlled by *fnr* a key transcription regulator in cells growing in anaerobic conditions and is up regulated under microaerophilic/anaerobic environments, was tested. *narX* expression was up regulated during biofilm growth, consistent with the development of microaerophilic conditions in the inner layers of *E. coli* biofilms (4.34 and 2 fold induction in 10 h and 55 h cultures).

### Expression of class Ib genes depends on the Fur and RpoS proteins

**Figure 6:**
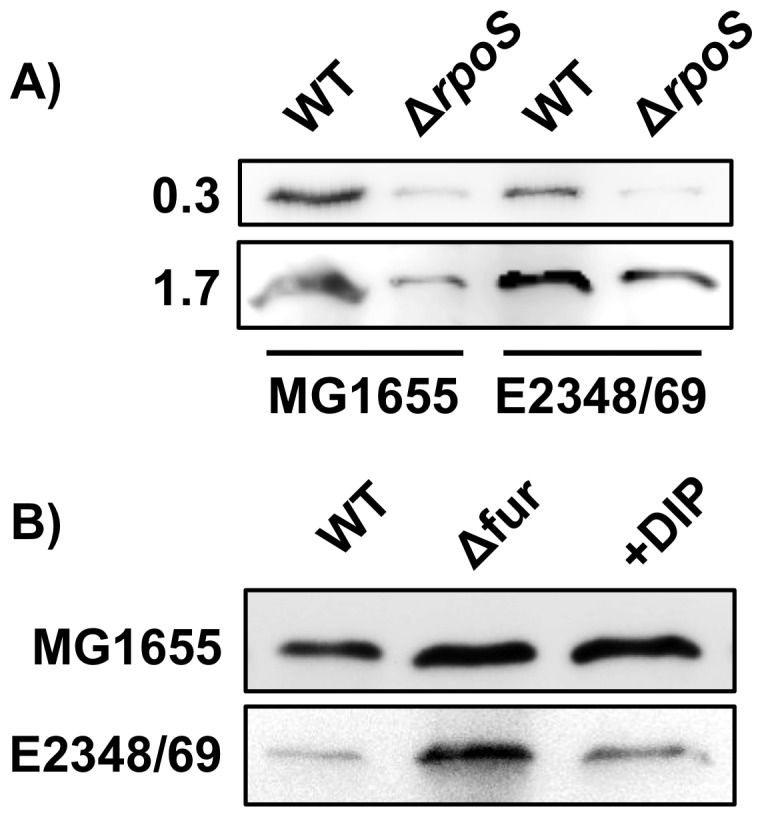
Class Ib RNR expression depend on the Fur and RpoS proteins. Western-blot analysis of the NrdF protein (class Ib RNR) in *E. coli* MG1655 and E2348/69 wild-type cells in A) *rpoS* mutant at stationary and exponential growth and B) *fur* mutant.

Several studies have described an increased expression of various genes affected by oxidative stress or nutrient starvation during biofilm formation [18]. Previous studies have suggested that the expression of class Ib RNR is up-regulated under nutrient starvation and oxidative stress [19]. Nevertheless the molecular mechanisms triggering their expression remain to be elucidated. Considering that our results showed that class Ib RNR was required and highly expressed during biofilm formation and especially during the initial steps, we decided to explore the factor(s) inducing the expression of this particular RNR class. RpoS has been shown to be upregulated in biofilms [20]. Furthermore, it has been evidenced in different global transcriptomic analysis that expression of class Ib RNRs is RpoS dependent [21], [22]. We decided to further correlate RpoS with class Ib RNR expression during biofilm formation. We performed RT-PCR analysis of *nrdE* expression on *E. coli* MG1655 and E2348/69 wild-type cells and their corresponding isogenic *rpoS* mutant strain ETS112 and ETS113 at different points on the exponential growth curve (OD_550_ = 0.3 and 0.8). Expression levels of the *nrdE* (class Ib) gene were found to be 2.61 and 9.44 fold higher (means of three different experiments) in the wild-type MG1655 cells than in the *rpoS* mutant derivative and 3.56 and 10.11 in the wild-type E2348/69 cells than in its *rpoS* mutant derivative (data not shown). To further confirm these results we used western-blot analysis to measure the NrdF expression (class Ib) in cells grown exponential and stationary growth phases in *E. coli* MG1655 and E2348/69 wild-type and *rpoS* mutants. In Fig6A is shown the dependence of class Ib on the presence of the RpoS protein. Both *E. coli* strains showed a reduced class Ib RNR expression in the *ΔrpoS* mutant compared to the wild-type strain at exponential and stationary growth phases, thus demonstrating the potential role of RpoS as an activator of the class Ib RNR and especially in biofilm formation where *rpoS* gene is highly induced [1], [20]. The precise mechanisms by which induces RpoS induces class Ib transcription needs to be further analysed.

It has been recently shown that oxidative stress induces the expression of class Ib genes (*nrdHIEF*) by inactivating of the ferric uptake regulator protein (FUR) [15]. This has also been extensively studied in other stress environments [23], [24]. A fur box was previously described in class Ib RNR promoter region [25]. This effect of class Ib activation through FUR inactivation is further supports the role class Ib RNR expression in biofilm formation where a high level of oxidative stress occurs [26]. This result was previously described for the *E. coli* MG1655 strain but we also tested if the pathogenic *E. coli* E2348/69 strain showed the same induction in the *nrdHIEF* expression as the commensal MG1655 strain [15], [25] and response to the FUR transcriptional regulator. Bioinformatics analysis of the *E. coli* E2348/69 class Ib promoter region revealed an 8 mer FUR box (CGTAATCA) at the same position as previously described in the *E. coli* MG1655 [25]. In Fig6B we described the repressor behaviour of the FUR transcriptional regulator on the class Ib expression. Band quantification shows that the addition of an specific iron-chelator DIP (see Material and methods) into the medium created a condition of iron deficiency and led the expression of class Ib (1.6 and 1.7 times in *E. coli* MG1655 and E2348/69 respectively) as well as the one found in the *fur* mutant (1.7 and 2.5 times in *E. coli* MG1655 and E2348/69 respectively) demonstrating the dependence of the class Ib transcription on the FUR regulatory protein as was also previously demonstrated in strain MG1655 [25], [27].

All together, it seems clear that class Ib RNR genes are highly induced during biofilm formation, as the cells cope with nutrient starvation (iron deficiency) and oxidative stress, and we can hypothesize that the molecular mechanism that triggers its expression is mediated directly through the Fur transcriptional factor and by the *rpoS* sigma factor which is highly induced under these metabolic conditions. Any direct participation of the RpoS protein or an indirect effect through another transcriptional factor remains to be investigated.

### Model for roles of ribonucleotide reductase classes in *Escherichia coli* biofilm formation

**Figure 7:**
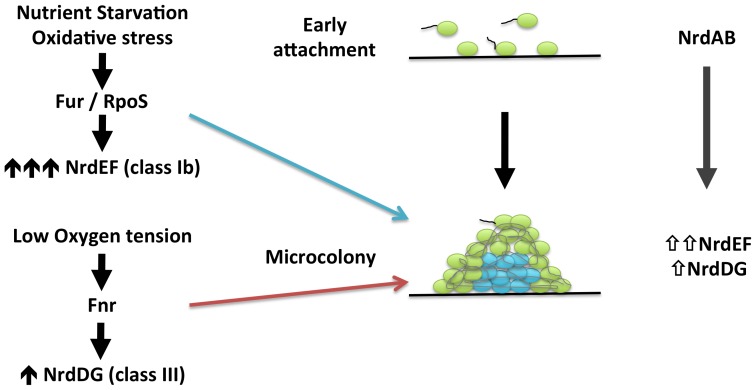
Proposed model for expression of ribonucleotide reductases in *E. coli* biofilm formation.

In this paper, we evidence that the hitherto cryptic class Ib RNR is expressed when *E. coli* cells form biofilms (see Fig.1, 4 and 5). Class Ia is active during standard laboratory growth conditions and also in planktonic cells, and down-regulated when cells switch to a biofilm state (Fig.5). Our results evidence an induction of RNR classes Ib and III during biofilm conditions. Hence, a physiological role for class Ib RNR is shown in these conditions for the first time (Figs.1,2,3,4,5). The need for simultaneous expression of RNR classes Ib and III has also been addressed. It is likely that during the early stages of attachment, planktonic cells express RNR class Ia (Fig.7). After attachment, *E. coli* strains and especially enteropathogenic *E. coli* (EPEC) isolates form microcolonies that develop into three-dimensional structures within which some regions are microaerophilic [6], [18]. Under these circumstances, with conditions of nutrient starvation and oxidative stress [26] class Ib expression is triggered, probably directly by the inactivation of the FUR protein or indirectly by RpoS, and is enzymatically functional. In these conditions, parts of this structure can result in a nutrient and oxygen limitation creating parts of microaerophilic areas. Class III RNR is fully enzymatically active under microaerophilic conditions [14] and it is highly expressed anaerobically by the FNR protein, as already described [12], [13]. Note that in *E. coli* E2348/69 strain, which forms more complex biofilms where more anaerobic environments can be created and in which expresses more *nrdD* protein (Fig.4) compared to the *E. coli* MG1655 strain which forms less structured biofilms. Other types of cells located at the inner parts of the biofilm might express class Ib RNR which can be active under these conditions [28] and favour its expression under oxidative stress and nutrient starvation as specified before.

For the first time, we have established a significant physiological role for class Ib and III RNR in *E. coli* and particularly in context of the formation of biofilms. Accordingly, class Ib and III RNR should be considered as a target for new anti-proliferative agents. Such agents might be useful in combined therapies to reduce and eliminate biofilms, making the chronic colonization/infection by certain pathogenic *E. coli* cells less virulent.

## Materials and Methods

### Bacterial strains, plasmids and culture conditions

All strains and plasmids used in this study are listed in Table1. *Escherichia coli* cells were grown in Luria-Bertani (LB) at 37°C. Antibiotics and chromogenic substrates were used at the following final concentrations: 50 µg ampicillin ml^−1^, 50 µg kanamycin ml^−1^, 30 µg chloramphenicol ml^−1^, 30 µg X-Gal ml^−1^. Bacterial growth was measured by reading OD_550_. In order to create iron-limiting conditions, the iron chelator 2,2′-dipyridyl (DIP) (SIGMA) was added at 150 µM to LB liquid cultures.

### Strain and plasmid construction

Plasmid DNAs were isolated using the QIAprep miniprep kit (Qiagen). PCRs were carried out using High-Fidelity PCR enzyme mix (Fermentas) according to the manufacturer's instruction. Other molecular biology techniques were carried out by standard procedures [29].

Different *nrd* mutant strains were constructed by introducing certain mutations (Table1) from the Keio collection *E. coli* strains [30] to the MG1655 and E2349/69 strains by P1 transduction [31] followed by selection of an appropriate drug resistance marker. All mutations were tested by PCR using a combination of gene-specific and transposon specific primers (Table2).

**Table 2:** Primers and probes used in this study.

Name	Sequence (5′→3′)	Application
EcoliDG-BHI-up	AAGGATCCGCCGTGAATGGAAG	Clonning
EcoliDG-BHI-lw	AAGGATCCTCGACATTCTGGTCGGTCAG	Clonning
Ecoli nrdR up	AAGGATCCCAGTCTTGCCGGTGTTTTCG	Clonning
Ecoli nrdR lw	AAGGATCCGCAACGCGTGTACTTCGGC	Clonning
Operon-Ib-up (BHI)	AAAGGATCCGCATGGTCGTACTCGCGTCC	Clonning
Operon-Ib-lw (BHI)	AAAGGATCCATTATTTCCCTGCTGCGGGTAGTG	Clonning
pBBR1-up	CATCGCAGTCGGCCTATTGG	Check-Clonning
pBBR1-lw	CACTTTATGCTTCCGGCTCG	Check-Clonning
M13 dir	GTTTTCCCAGTCACGAC	Check-Clonning
M13 rev	CAGGAAACAGCTATGAC	Check-Clonning
k1	CAGTCATAGCCGAATAGCCT	Check-Clonning
kt	CGGCCACAGTCGATGAATCC	Check-Clonning
k2	CGGTGCCCTGAATGAACTGC	Check-Clonning
EcA-lw	TCAGATCTTACATGCGCCGC	Reverse transcription
EcD-lw	GGCTTCATCGCCTTTTGCTTCC	Reverse transcription
EcR-lw	GCGCGATCTCTTCGCCAAATTC	Reverse transcription
EcE-lw	AAAGTGCACAGGAGACGCAG	Reverse transcription
EcGAP-lw	GATGATGTTCTGGGAAGCGC	Reverse transcription
PnrdA BamHI up	AAAGGATCCATCATTTTCTATAAGACGG	Promoter-probe clonning
PnrdA ClaI lw	AAAATCGATCACCAGCAGATTCTGATTCATG	Promoter-probe clonning
PnrdD BamHI up	AAAGGATCCTTGAGGCTGTCTGGTGGTTAC	Promoter-probe clonning
PnrdD ClaI lw	AAAATCGATGCACTTTGCAGCCGTCTCG	Promoter-probe clonning
PnrdH BamHI up	AAAGGATCCAAAAATGATAATAAATACGCG	Promoter-probe clonning
PnrdH ClaI lw	AAAATCGATGTAAATAGTAATGCGCATG	Promoter-probe clonning

To construct plasmids pETS154, pETS155, pETS156 and pETS157 the *nrdHIEF* (class Ib), *nrdDG* (class III) and *nrdR* operons were cloned into plasmids pBluescriptSK(+) and pBBR1MCS-5 under the control of their respective native promoters (Table1 and 2).

Comparison of the growth curves of the wild type, mutants and complemented strains did not show any significant differences (data not shown).

### Biofilm formation

Biofilm formation was tested on polyvinyl chloride (PVC) 96-well plates using crystal violet staining as previously described [32]. Briefly, strains from an overnight culture were inoculated at an OD_550_ of 0.01 in LB with 0.2% glucose into wells of PVC microtiter plates (COSTAR) and incubated at 37°C, without agitation, for 48 h. The attached cells were stained with 0.1% crystal violet and biofilm formation was quantified by dissolving the CV in ethanol and measuring the absorbance at 570 nm (A_570_).

To study biofilm development on glass surface, we followed basically the method described previously by O′Toole [33]. 3 mL aliquot of diluted culture was added to a borosilicate glass tube containing a sterile 18×18 mm glass cover slip (Menzel-Gläser) and incubated at 37°C for 48 h. Excess of broth was removed and washed three times in PBS and attached cells were fixed in methanol and stained with 0.1% crystal violet. Biofilm was quantified by dissolving the attached cells in ethanol and absorbance was measured as described above.

### RNA preparation and quantitative RT-PCR

Total RNA was extracted from *E. coli* cells using the RNeasy Mini kit (Qiagen) according the manufacturer's instruction. RNA quantity was measured using a NanoDrop spectrophotometer (ND-1000, NanoDrop). RNA samples were treated for DNA digestion with DNase I (Turbo DNA-free, Ambion) and 1 µg was reverse-transcribed with the SuperScript® III Reverse transcriptase kit (Invitrogen). Real-time PCR measurements were carried out using TaqMan primers and probes, and detection was performed using and ABI Prism 7700 Sequence Detection System from Applied Biosystems as described previously [11]. The 16S rRNA sequence was used as an internal standard.

### Construction of GFP fusions

Transcriptional fusions in *E. coli* were constructed by PCR amplification of the promoter region of the *nrdA* (706 bp), *nrdH* (268 bp) and *nrdD* (462 bp) and cloning of these regions upstream of the promoterless *gfp* gene in pETS130-GFP [34]. Primers used for these cloning are described in Table2. Sequencing and PCR analysis confirmed the orientation and correctness of the inserts. GFP fluorescence was measured on an FLx800 Fluorescence microplate reader (BioTek). All assays were averages of at least three independent trials.

### Detection of NrdE by Western blot analysis

SDS-page and immunobloting were performed as described previously [34]. Briefly, protein crude extracts were extracted with the BugBuster® extraction reagent (Novagen) from *E. coli* culture samples. 5 µg of protein from each condition were resolved on a 7.5% polyacrylamide gel and transferred to PVDF membranes (Immun-Blot™ PVDF membranes, Bio-Rad). The immunodetection of proteins was performed using rabbit polyclonal antibodies against NrdF protein [35] at dilution 1/1000 (Agrisera, Sweden). The detection of primary antibodies was performed using donkey anti-rabbit (Bio-Rad) horseradish-peroxidase-conjugated secondary antibodies at 1/50000 dilution, and visualized using the Amersham™ ECL™ Prime western blotting reagent (GE Healthcare) according the manufactured's protocol. The membrane was visualized and analyzed with an ImageQuant™ LAS4000 mini (GE Healthcare). The polyclonal antibodies cross-reacted with few proteins which was used as internal control for equal protein loading.
